# Aesthetic perception of visual textures: a holistic exploration using texture analysis, psychological experiment, and perception modeling

**DOI:** 10.3389/fncom.2015.00134

**Published:** 2015-11-04

**Authors:** Jianli Liu, Edwin Lughofer, Xianyi Zeng

**Affiliations:** ^1^College of Textile and Clothing, Jiangnan UniversityWuxi, China; ^2^Department of Knowledge-Based Mathematical Systems/Fuzzy Logic Laboratorium Linz-Hagenberg, Johannes Kepler University LinzLinz, Austria; ^3^The ENSAIT Textile Institute, University of Lille Nord de FranceRoubaix, France

**Keywords:** visual texture, aesthetic emotion, texture analysis, psychological experiment, dimension reduction, perception modeling, layered model architecture

## Abstract

Modeling human aesthetic perception of visual textures is important and valuable in numerous industrial domains, such as product design, architectural design, and decoration. Based on results from a semantic differential rating experiment, we modeled the relationship between low-level basic texture features and aesthetic properties involved in human aesthetic texture perception. First, we compute basic texture features from textural images using four classical methods. These features are neutral, objective, and independent of the socio-cultural context of the visual textures. Then, we conduct a semantic differential rating experiment to collect from evaluators their aesthetic perceptions of selected textural stimuli. In semantic differential rating experiment, eights pairs of aesthetic properties are chosen, which are strongly related to the socio-cultural context of the selected textures and to human emotions. They are easily understood and connected to everyday life. We propose a hierarchical feed-forward layer model of aesthetic texture perception and assign 8 pairs of aesthetic properties to different layers. Finally, we describe the generation of multiple linear and non-linear regression models for aesthetic prediction by taking dimensionality-reduced texture features and aesthetic properties of visual textures as dependent and independent variables, respectively. Our experimental results indicate that the relationships between each layer and its neighbors in the hierarchical feed-forward layer model of aesthetic texture perception can be fitted well by linear functions, and the models thus generated can successfully bridge the gap between computational texture features and aesthetic texture properties.

## Introduction

Texture is ubiquitous. It contains important visual information about an object and allows us to distinguish between animals, plants, foods, and fabrics. This makes texture a significant part of the sensory input that we receive every day. In the visual arts, texture is the perceived surface quality of a work of art. It is an element of two- and three-dimensional designs and is distinguished by its perceived visual and physical properties (Graham and Meng, 2011). From the research point of view, textures are classified into tactile and visual textures. The former, also known as actual textures or physical textures, are actual surface variations (Elkharraz et al., 2014), including, but not limited to, fur, wood grain, sand, and the smooth surfaces of canvas, metal, glass, and leather (Skedung et al., 2013). Physical texture is distinguished from visual texture by a physical quality that can be felt by touch (Manfredi et al., 2014). Visual texture is the illusion of physical texture. Every material has its own visual texture. Photographs, drawings, and paintings use visual texture to portray their participant matter both realistically and with interpretation (Guo et al., 2012). Above all, visual scientists have realized that the rich resource they are provided with by artists in the form of textures is worthy of scientific study (Zeki, 2002).

The challenge in aesthetic perception of visual textures and art is to understand the aesthetic emotion and judgment that are evoked when we experience beauty. To evaluate and explain beauty in science, models of aesthetic perception and judgment have been proposed in cognitive psychology and information science. According to the information-processing stage model of aesthetic processing, five stages-perception, explicit classification, implicit classification, cognitive mastering, and evaluation are involved in aesthetic experiences (Leder et al., 2004).

To discriminate between aesthetically pleasing and displeasing images, Datta et al., employed support vector machines and classification trees to perform explicit classification, and applied linear regression to polynomial terms of features to infer numerical ratings of aesthetics (Datta et al., 2006). Additionally, Datta et al., developed multi-category classifiers to recognize coarse-grained aesthetic categories and used support vector machines to predict fine-grained aesthetic scores (Datta et al., 2006). Jiang et al. used two model built algorithms to study automatic assessment of the aesthetic value in consumer photographic images (Jiang et al., 2010). Cela-Conde et al. pointed out that investigating the cognitive and neural underpinnings of aesthetic appreciation by means of neuro-imaging has yielded a wealth of fascinating information (Cela-Conde et al., 2011). Toet et al. explored the effects of various spatiotemporal dynamic texture characteristics on human emotions (Toet et al., 2011). Using structural equation modeling, Leder et al. explored aesthetic perception by analyzing expertise-related differences in the aesthetic appreciation of classical, abstract, and modern artworks (Leder et al., 2012). Simmons explored the relationship between color information and the emotions they induced by measuring along two affective dimensions, namely pleasant-unpleasant, and arousing-calming (Simmons, 2012).

In their research, Cela-Conde et al. discussed adaptive and evolutionary explanations for the relationships between the default mode network and aesthetic networks, and offered unique input to debates on the interaction between mind and brain (Cela-Conde et al., 2013). Reviewing from definitional, methodological, empirical, and theoretical perspectives of human aesthetic preferences, Palmer et al. concluded that visual aesthetic response can be studied rigorously and meaningfully within the framework of scientific psychology (Palmer et al., 2013). The research of Bundgaard addressed the phenomenology of aesthetic experience, which showed why and how aesthetic experience should be defined relative to its object and the tools for meaning-making specific to that object and not relative to the feeling (Bundgaard, 2014). Chatterjee and Vartanian reviewed recent evidence that approves aesthetic experiences emerge from the interaction between sensory–motor, emotion–valuation, and meaning–knowledge neural systems (Chatterjee and Vartanian, 2014). In experiment, Elkharraz et al. designed and manufactured 3D tactile textures with predefined affective properties, and used mixing algorithms to synthesize 48 new tactile textures that were likely to score highly against the predefined affective properties (Elkharraz et al., 2014).

However, surprisingly little funded research has been conducted on the emotional qualities and expectations associated with specific textures. In 2007, the project named “Measuring Feelings and Expectations Associated with Texture” (SynTex) was supported by the European Commission within the sixth framework program. SynTex was coordinated by Profactor GmbH and conducted in collaboration with six other research institutes in the European Union. In fact, SynTex is the only project to have ever attempted to measure, model and predict the psychological effects of texture. Thumfart et al. summarized the outcomes of this project (Thumfart et al., 2011). A further outcome is in the work of Groissboeck, which focused on synthesizing textures for predefined, desired emotions described by a numerical vector in aesthetic space (Groissboeck et al., 2010). We build upon this research, but go a step further in terms of significantly enhanced texture analysis, feature selection, and layered model-building for better interpretability, while achieving improved accuracy in the prediction of several core adjectives that define the aesthetic space. Expanding the aesthetic space used in Thumfart et al. (2011), we introduced two new adjectives in our experiments.

After reviewing related work in Section Introduction, we present the four different categories of low-level features that were extracted to objectively represent the visual textures in Section Materials and Methods. Further, we describe feature selection using Laplacian Score to reduce the complexity of the aesthetic perception model. Section Results and Discussion summarizes the semantic differential rating experiment, in which we collected aesthetic perceptions from participants with selected textural stimuli. We describe the modeling approaches in Section Results and Discussion; Section Conclusions conclude the paper.

## Materials and methods

### Selected textural stimuli

**Figure 1:**
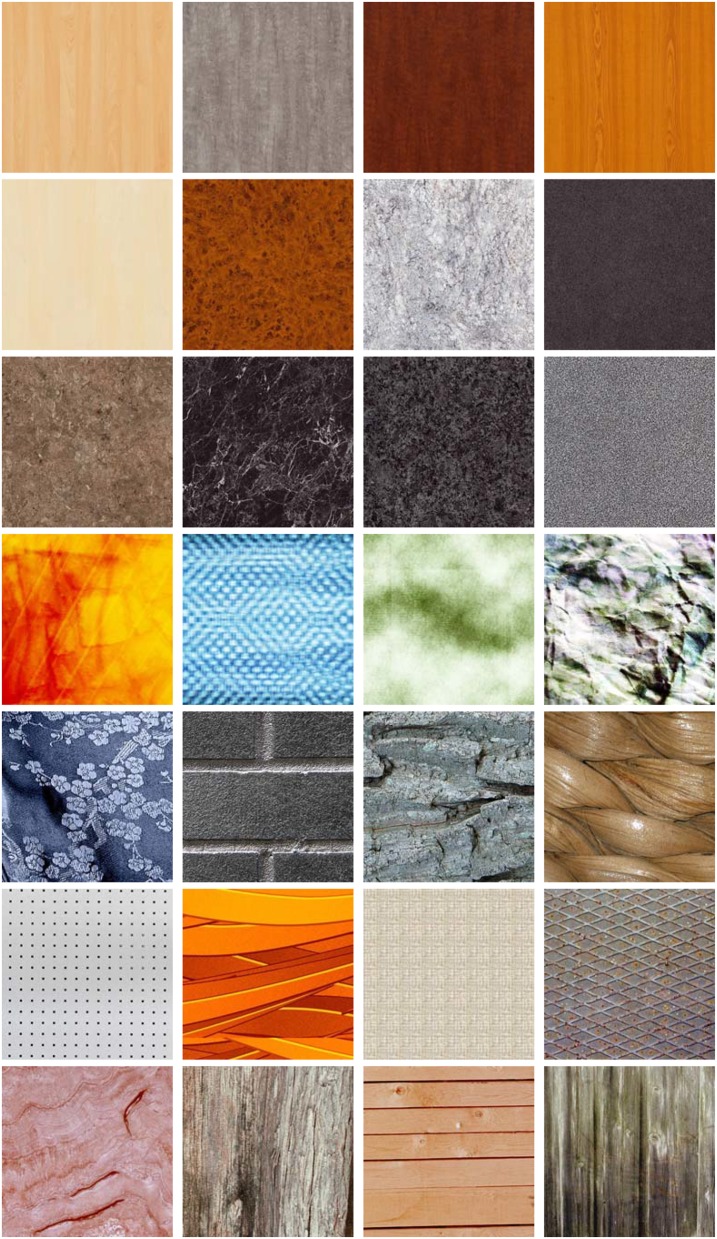
**Some examples of visual textures from the SynTex collection**.

The visual texture database of stimuli used in our experiment consists of 151 selected high-resolution textural images, which are also the experiment materials used in SynTex project. This database is the Supplementary Material of the paper published by Thumfart et al., in the proceedings of the 13th international conference on Computer Analysis of Images and Patterns (CAIP 2009) (Thumfart et al., 2009). The project SynTex is by far outdated and the link that provides the visual texture database has been closed. The used visual textures for our study can be sent to readers upon request via email or dropbox exchange. Readers can contact us by using the email addresses given in the affiliations. It includes natural, artificial, regular and stochastic textures in the textural stimuli, which were selected from various texture databases. In detail, 73 textures were chosen from the Brodatz texture album, 69 from the Outex texture database, 25 from the UIUCTex database, 12 from the USC-SIPI image database, and 64 from the VisTex database. Since the original sizes of the textures selected from different database varied, they were resized to a resolution of 480 × 480 pixels. Some examples of visual textures from the SynTex database are shown in Figure 1.

In the SynTex database, some textures are artificial and synthetic, some others are natural. So a nice diversity of different sorts and types of textures is given.

### Texture analysis

Texture analysis refers to the characterization of image regions by their textural content (Karu et al., 1996). Texture analysis attempts to quantify intuitive qualities described by terms such as rough, smooth, silky, and bumpy as functions of the spatial variations in pixel intensities (Guo et al., 2012). Texture analysis is used in a variety of applications, and can be helpful when objects in an image are better characterized by their textures than by intensity or traditional thresholding techniques (Bharati et al., 2004).

In our experiment, four different texture analysis methods are employed to extract statistical characteristics from visual textures, which were then categorized into color and statistical features, and perceptual and frequency-domain energy-based features. In total, we initially derived a set of 106 features for each texture image.

#### Color characteristics

Colors play an important role in deciding what we like or dislike, because they evoke complex psychological reactions and give rise to relevant feelings (Ou et al., 2004a,b). In addition to the studies of Simmons (2012) mentioned in the introduction, there is growing interest in the understanding of human feelings in response to seeing colors and colored objects, which are also called “color emotions” in psychology (Lucassen et al., 2011). Experimental results show that the emotional responses to warm/cool, heavy/light, and active/passive are consistent across cultures, but that the like/dislike scale exhibits some differences (Ou et al., 2012). Visual perception of some emotions can be linked to different colors (Augello et al., 2013). Regression analysis is usually applied before product color design to reveal the relationships between human responses on these scales and the underlying color appearance attributes, such as lightness, chroma, and hue (Hanada, 2013; Man et al., 2013).

Six color features were computed from HSV (hue-saturation-value) space to describe each visual texture as the ones used in the work of Romani et al. (2012). In detail, average, and standard deviation of the HSV color matrix elements were calculated after conversion of each texture image from RGB to HSV color space.

#### Gray level co-occurrence matrix characteristics

If texture is the dominant information in a small area, then this area has statistically a wide variety of discrete textural features (Baraldi and Parmiggiani, 1995). The simplest texture analysis method uses statistical features computed from histograms. Haralick et al., went a step further and proposed a gray-level co-occurrence matrix (GLCM) in which the relative positions of pixels with respect to each other are considered as well (Haralick et al., 1973; Roberti et al., 2013). Given a spatial relationship between pixels in a texture, such a matrix represents the joint distribution of gray-level pairs of neighboring pixels (Davis et al., 1979). Thus, a considerable amount of information can be obtained by modifying the orientation θ or distance *d* between pixels, where *d* specifies the distance between the pixel of interest and its neighbor, and θ gives the direction from the pixel of interest to its neighbor. If either θ or *d* is set, one GLCM is generated. From each GLCM, four statistical characteristics called contrast, correlation, energy, and homogeneity can be calculated.

To research the effect of distance and orientation on statistical features, we extracted 16 GLCMs, choosing the distance from the set *d* = {2, 4, 6, 8} and the orientation from θ = {0°, 45°, 90°, 135°}. Use of these orientation angles, restriction to 135° is inspired by Haralick et al. They have been employed in many published statistical representations of textures and are deemed to provide sufficient information for building gray-level co-occurrence matrices. In total, we extracted 64 statistical features for each computed GLCM.

#### Tamura texture features

In color emotion research, an object usually has a uniform color. However, this is rarely the case for real-life objects. Therefore, the effect of texture on color emotion should be extended. Tamura, Mori, and Yamawaki found in psychological studies that humans respond best to coarseness, contrast, and directionality, and to lesser degrees to line-likeness, regularity, and roughness (Tamura et al., 1978). In most cases, only the first three Tamura features capture the high-level perceptual attributes of a texture well and are useful in visual art appreciation (Castelli and Bergman, 2002). Thus, in contrast to statistical data measures, Tamura texture features seem well suited to capture the emotional perception of visual textures. In our experiment, coarseness, contrast, and directionality were calculated as characteristics that represent the psychological responses to visual perception.

#### Wavelet-based energy texture features

Wavelets have been successfully used as an effective tool to analyze texture information, as they provide a natural partitioning of the image spectrum into multi-scale and oriented sub-bands via efficient transforms (Brooks et al., 2001). Furthermore, wavelets are used in major image compression standards and are prominent in texture analysis (Dong and Ma, 2011). Wavelet-based energy features can be extracted as frequency features in conjunction with other spatial features to capture visual texture information. The basic idea underlying the wavelet energy signature is to generate textural features from wavelet sub-band coefficients or sub-images at each scale after wavelet transformation (Liu et al., 2011). Assuming that the energy distribution in the frequency domain identifies texture, we used *L*^1^ and *L*^2^ norms as measures in our work. More specifically, we calculated *L*^1^ and *L*^2^ norms from the high-frequency sub-bands of the first four levels that were proposed by Do and Vetterli (2002). To describe the quality of the information included in each sub-image to be reconstructed with the corresponding wavelet coefficients, we also calculated the Shannon entropy of each high-frequency sub-band.

When a texture image is decomposed at level *j* using a 2D discrete wavelet base, 3 *j* sub-bands are generated. Then 6 *j* energy signatures and 3 *j* entropy signatures are extracted. Since we decomposed each texture image into 4 levels, we extracted 36 wavelet signatures from each texture image.

### Feature selection using the laplacian score

The goal of feature selection is to select the best features from a set of features that not only achieve the maximum prediction rate but can also reduce the complexity of model building (Vapnik, 1998). All feature selection approaches can be applied in either supervised or unsupervised mode (Chandrashekar and Sahin, 2014). In supervised mode, each training sample is described by a vector that consists of feature values with a class label. The class labels are used to guide the search process toward the optimal feature subset. In unsupervised mode, the training samples are not labeled, and thus feature selection is more difficult (Tabakhi et al., 2014). However, this mode provides more general information which can be used by an arbitrary model architecture.

Predicting aesthetic emotions linked to visual textures is a typical example of data mining, where the inputs are low-level texture features and the outputs are aesthetic properties of visual textures. The aesthetics properties used as outputs for modeling are not labeled by strings or 1-0 codes (class labels), as is usual in classification problems, but by discrete real decimal numbers. Ideally, as discussed above, the feature selection method should be independent of the chosen model architecture and also of the hierarchical layered structure (Breiman et al., 1993). Furthermore, we sought to optimize the information content of the feature space while reducing its complexity, with the aim of obtaining one unique reduced set with good interpretation capability.

In a kind of filter selection stage, we thus focused on an unsupervised feature selection scheme called Laplacian Score (LS). LS is a relatively recent unsupervised method for selecting top features (He et al., 2005). It is able to reduce truly redundant and correlated information content of the extracted features—note that only truly redundant features can be discarded without significant information loss (see Guyon and Elisseeff, 2003). In detail, firstly a nearest-neighbor graph was constructed for the original feature set. Secondly, the Laplacian scores for all features in the original feature set were computed using the LS algorithm. Thirdly, the features were ranked according to their Laplacian scores in ascending order. Finally, the last *d* features were discarded, and the feature set was updated with only the remaining features.

### Psychological experiments and perception modeling

Aesthetic experiences are very common in modern life, even we don't deliberately care about them. There is yet no scientifically comprehensive theory that explains what constitutes such experiences. As mentioned in the Introduction section, several scientific methods have been used to explore the complex systems that involve in aesthetic experiences. Except for measurements of physiological signals using bio-sensors, psychological experiments are also important tools in exploring cognitive challenges of aesthetic experience and judgments. This section describes the semantic differential rating experiment that was conducted to collect their aesthetic perceptions of visual textures from 10 male and 10 female subjects. The aesthetic properties were assigned to three different layers of the proposed aesthetic perception model.

#### Definitions of the aesthetic properties

Before the semantic differential experiment, we had to select and define the aesthetic properties. Which types of aesthetic properties should be defined and how many pairs of aesthetic antonyms should be selected are hot research topics in semantic analysis. The definition of the eight core adjectives as shown in Table 1 has been derived from the findings in Levinson (2006) which emphasized that six of these define the aesthetic core space. The two additional ones (dark-light and disordered-harmonious) were considered because of the contents of the textures selected for experiment and the suggestions coming from the 20 subjects.

**Table 1:** **Eight pairs of aesthetic properties are divided into three layers**.

**Aesthetic property**	**Emotion layer**	**Aesthetic property**	**Emotion layer**
Warm-cold	Affective layer	Inelegant-elegant	Judgment layer
Rough-smooth	Affective layer	Simple-complex	Judgment layer
Dark-light	Affective layer	Artificial-natural	Judgment layer
Disordered-harmonious	Judgment layer	Like-dislike	Emotional layer

Before the semantic differential experiments, we explained the meaning of each pair of aesthetic antonyms to the 20 participants and showed them some typical samples. In experiment, we emphasized that these samples were not their only references. We further suggested that knowledge about and preference for—or even prejudice against—some types of visual texture they would encounter should also be considered.

As shown in Table 1, the 8 pairs of aesthetic antonyms are also assigned to three emotion layers defined in Thumfart's work. In fact, the 8 pairs of aesthetic antonyms are assigned to effective, judgment or emotional layer by the 20 subjects after surveying 100 persons in 3 days. The logic relationships between these emotion layers are explained in Section Aesthetic Perception Model of Visual Textures.

#### Semantic differential experiment

Semantic differential experiments are commonly used to explore perceptual and emotional dimensions of visual art and music. In our case, a semantic differential experiment was carried out—with the approval of the ethical committee of Jiangnan University for experiments with human participants—to collect participant ratings for the eight aesthetic properties defined in Table 1. In the semantic differential experiment, 20 highly motivated Jiangnan University undergraduate students (aged 19–23) served as participants to rate 151 visual textures in terms of eight aesthetic antonyms. Before experiment, we introduced our research purpose, experimental procedures, and how long it takes to participate to all participants, and provided a written informed consent form to each participant.

After signing a written informed consent form, each participant enrolled for at least 5 daily sessions of 2 h and received payment. The purpose of the experiments was concealed from all participants, and they were trained to use a program we developed called Texture Aesthetic Annotation Assistant to rate the defined aesthetic properties. In each test, participants briefly (300 ms) viewed one visual texture, which was followed immediately by a perceptual mask (200 ms) presented at the same location. The viewing distance was 75 cm (screen to participant). After training, the 20 participants participated in the semantic differential experiment in our lab at their own leisure.

The participants operated the Texture Aesthetic Annotation Assistant which automatically displayed the texture and stored the ratings in a e. A visual texture and a rating bar were shown in the center and at the bottom of the display. The subject could drag the scrollbar to rate the texture according to the labeled aesthetic antonyms (placed at opposite ends of the scrollbar), and the eight pairs appeared sequentially as listed in Table 1. Rather than the seven point rating scale, we used a continuous rating scale within the interval [−100, 100] (Chuang and Chen, 2008). This kind of rating method is useful to build a continuous regression model with sufficiently fine granularity.

In the semantic differential experiment, each participant randomly evaluated each texture five times, and the ratings for each texture were stored in a text file. After completion of the semantic differential experiment, the ratings for each visual texture evaluated by the 20 participants (i.e., 100 ratings per texture) were averaged and used as final ratings to build a prediction model for aesthetic emotions (see below).

As the aim of this research was to gain general insights and explore potential relationships between human texture perception and low-level features of visual textures, we did not use individual experimental data to build an individual model for each subject, but created a general model that may be valid for a wider range of applications and purposes and reduces development costs.

#### Aesthetic perception model of visual textures

Axelsson summarized five theoretical models that are most important for the development of psychological aesthetics: (1) Berlyne's Collative-Motivational Model, (2) the Preference-for-Prototypes Model, (3) the Preference-for-Fluency Model, (4) Silvia's Appraisal-of-Interest Model, and (5) Eckblad's Cognitive-Motivational Model (Axelsson, 2007). However, these five models were developed in theoretical psychology and can hardly be explained in information-processing and mathematical terms because the input factors are specific human emotions that cannot be quantified. The hierarchical layer structure of these models, however, provides a reference for our work, and some aesthetic properties involved there are also helpful to us.

**Figure 2:**
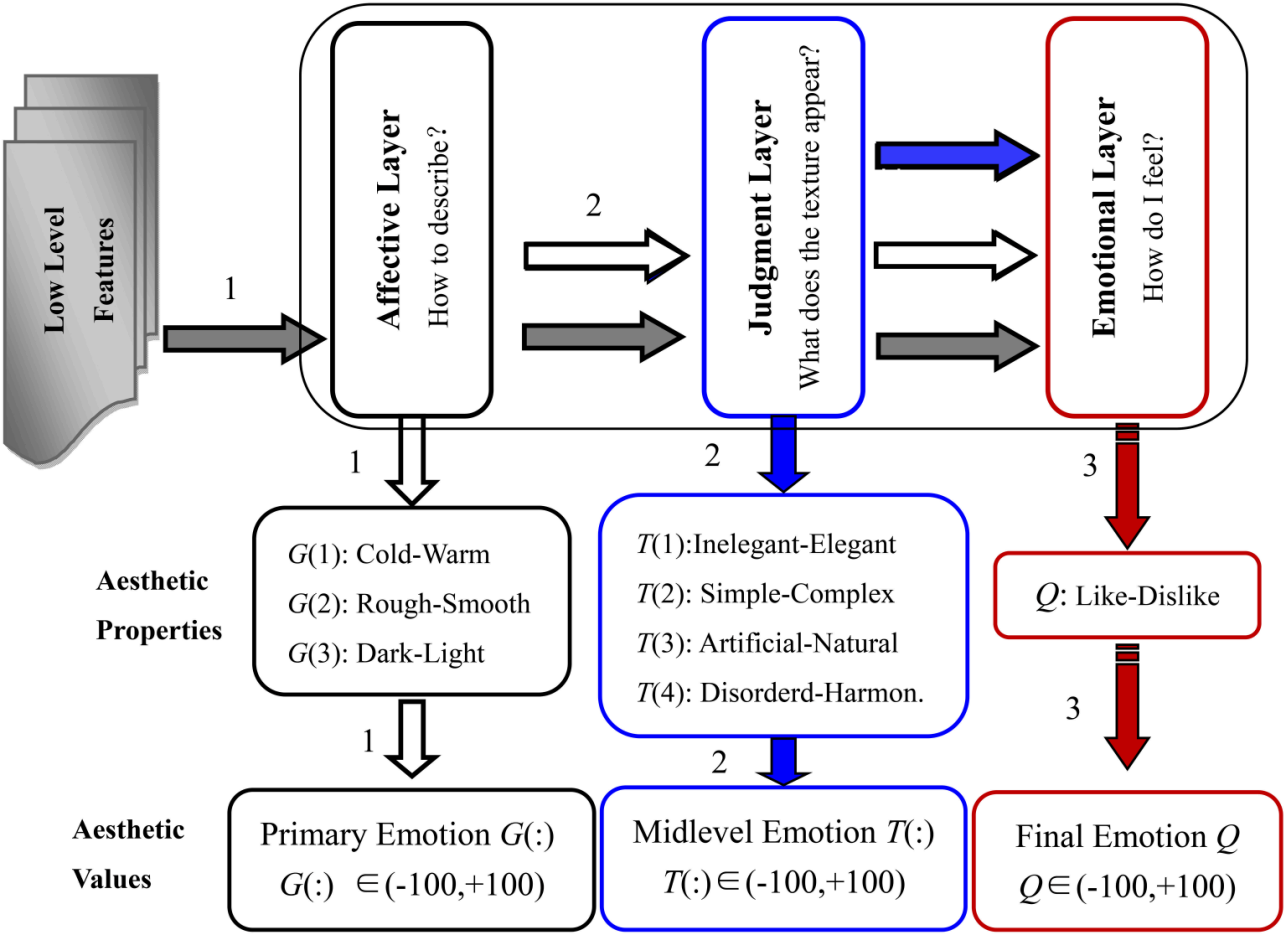
**Structure of the hierarchical feed-forward model of aesthetic perception of visual texture, the horizontal arrows indicate input flows to the different layers: e.g., the inputs to the judgment layer are low-level features (gray arrow) plus properties of the affective layer (white arrow)**.

Achievements in neuroaesthetics are the most important basis for building a hierarchical structure of aesthetic perception, especially the research of Ishizu and Zeki provides powerful support (Ishizu and Zeki, 2013). Also, Thumfart et al. applied a similar hierarchical layer structure, in which we extended with two additional properties, “dark-light” and “disordered-harmonious.” The structure of the hierarchical feed-forward model of aesthetic texture perception is shown in Figure 2.

In the hierarchical feed-forward model, the function of the affective layer is to complete the descriptions of the general and primary physical properties of the visual texture. Thus, the aesthetic antonyms selected for the affective layer are used to capture the primary emotions when we first skim the visual textures. In the judgment layer, the selected aesthetic antonyms should describe higher-level and more aggregate properties that are in part anchored in the subconscious, especially those induced after statistical and logical judgment. The emotions we feel when interacting with the textures are described in the emotional layer. The aesthetic antonyms selected for the emotional layer should describe the overall feelings people have and wish to express.

#### Building an aesthetic perception model

Traditional machine learning techniques such as neural networks and support vector regression are useful prediction tools. However, they become completely impractical when interpretability of the implicit relations between low-level features and core adjectives is desired, because they are black boxes and cannot provide any meaningful and understandable insights. Hence, we propose a hierarchical feed-forward layer model of aesthetic texture perception with high interpretability that combines neuroaesthetics and information processing theory. In the layered structure model, each layer has a set of interpretable aesthetic antonyms.

As illustrated in Figure 2, there are three perception channels similar to neural circuits in neuroscience. In the first channel, the low-level texture features are used to model the aesthetic properties of the affective layer. In the second channel, the properties of the judgment layer are modeled using low-level features and aesthetic properties of the affective layer. Finally, the properties of the emotional layer are built accordingly by inputs from all previous layers and low-level features.

We set $M_{p}=\left\{A_{i}^{p},B_{j}^{p},C_{k}^{p},\ldots \right\}$ as the low-level feature set of the *p*^*th*^ visual texture, where *i* = 1, 2…*n*, *j* = 1, 2…*s*, *k* = 1, 2…*t* represents the number of different texture feature subsets *A, B, C*, etc. The aesthetics values of the affective layer, the judgment layer and the emotional layer for the *p*^*th*^ visual texture are represented by $G_{p}=\left\{g_{1}^{p};g_{2}^{p};g_{3}^{p}\right\}$, $T_{P}=\left\{t_{1}^{p};t_{2}^{p};t_{3}^{p};t_{4}^{p}\right\}$, and $Q_{p}=\left\{q^{p}\right\}$, respectively. Considering the ideas conveyed in Figure 2, we employ six activation functions to construct the three perception channels.

The perception model of the affective layer is given by:

(8) $$\begin{array}{l} G=F_{1}\left(M\right)+R_{0} \end{array}$$

that of the judgment layer by:

(9) $$\begin{array}{l} T=F_{2}\left(M\right)+F_{3}\left(G\right)+R_{1} \end{array}$$

and that of the emotional layer by:

(10) $$\begin{array}{l} Q=F_{4}\left(M\right)+F_{5}\left(G\right)+F_{6}\left(T\right)+R_{2} \end{array}$$

where *F*_1_, *F*_2_, *F*_3_, *F*_4_, *F*_5_, and *F*_6_ are the six activation functions that can be linear or non-linear, and *R*_0_, *R*_1_, and *R*_2_ refer to the emotion thresholds. The symbol “+” indicates emotions accumulated through different perception stages. Note that in our model-building cycles (as explained in the Results section), a particular set of activation functions best suited to the problem at hand is automatically applied. A standard procedure consists of a weighted linear combination of these activation functions where the weights are derived by least-squares optimization to obtain an optimal solution within a closed analytical formula, see Ljung (1999) or Lughofer (2011).

When aesthetic emotions are predicted for new incoming textures, the adjectives in the affective layer G(1), G(2), and G(3) are predicted using the low-level feature set stored in M and applying the activation function *F*_1_. Next, the adjectives in the judgment layer T(1), T(2), T(3), and T(4) are predicted using the low-level feature set M and the predicted adjective values G(1) to G(3) by applying activation functions *F*_2_ and *F*_3_. Finally, the emotional layer adjective (“like-dislike”) is predicted using the low-level feature set M, the predicted adjective values G(1) to G(3) and the predicted values T(1), T(2), T(3), and T4) by applying activation functions *F*_4_, *F*_5_, and *F*_6_. Alternatively, if adjective values for G(1) to G(3) and/or T(1) to T(4) are already given by humans, these can be used in place of the predictions.

## Results and discussion

### The selected top features

After feature selection, the original 106-D features were ranked according to their Laplacian scores. The first and most important 15 features are listed in Table 2.

**Table 2:** **Feature list after selection using the Laplacian score**.

**ID**	**Laplacian score**	**Category**	**Parameters**	**Name**
*f*_1_	0.9742	Color characteristics	Mean of saturation	Mean of saturation
*f*_2_	0.9101	GLCMs	*d* = 8, θ = 45°	Contrast
*f*_3_	0.8995	GLCMs	*d* = 6, θ = 45°	Contrast
*f*_4_	0.8855	GLCMs	*d* = 8, θ = 135°	Contrast
*f*_5_	0.8785	GLCMs	*d* = 8, θ = 90°	Contrast
*f*_6_	0.8778	GLCMs	*d* = 4, θ = 45°	Contrast
*f*_7_	0.8690	Tamura texture		Coarseness
*f*_8_	0.8656	Tamura texture		Directionality
*f*_9_	0.8551	Wavelet-based energy	horizontal sub-band at level 1	*L*^2^ norm
*f*_10_	0.8434	Wavelet-based energy	vertical sub-band at level 1	*L*^2^ norm
*f*_11_	0.8434	Tamura texture		Contrast
*f*_12_	0.8427	GLCMs	*d* = 8, θ = 45°	Homogeneity
*f*_13_	0.8367	GLCMs	*d* = 8, θ = 135°	Homogeneity
*f*_14_	0.8306	GLCMs	*d* = 6, θ = 45°	Homogeneity
*f*_15_	0.8282	Wavelet-based energy	horizontal sub-band at level 1	*L*^1^ norm

The first 15 texture features are listed in Table 2 according to their Laplacian score in ascending order. The first feature is the mean saturation, which is extracted from the HSV space. The number of contrast and homogeneity calculated using GLCMs is eight, which accounts for 53%. The ranks of coarseness and directionality are the seventh and the eighth, respectively. The ranks of the wavelet-based energy texture features (*L*^1^ norm and *L*^2^ norm) calculated from the horizontal and vertical sub-band at the first level are the ninth, the tenth and the fifth.

### Visualization of the selected features

**Figure 3:**
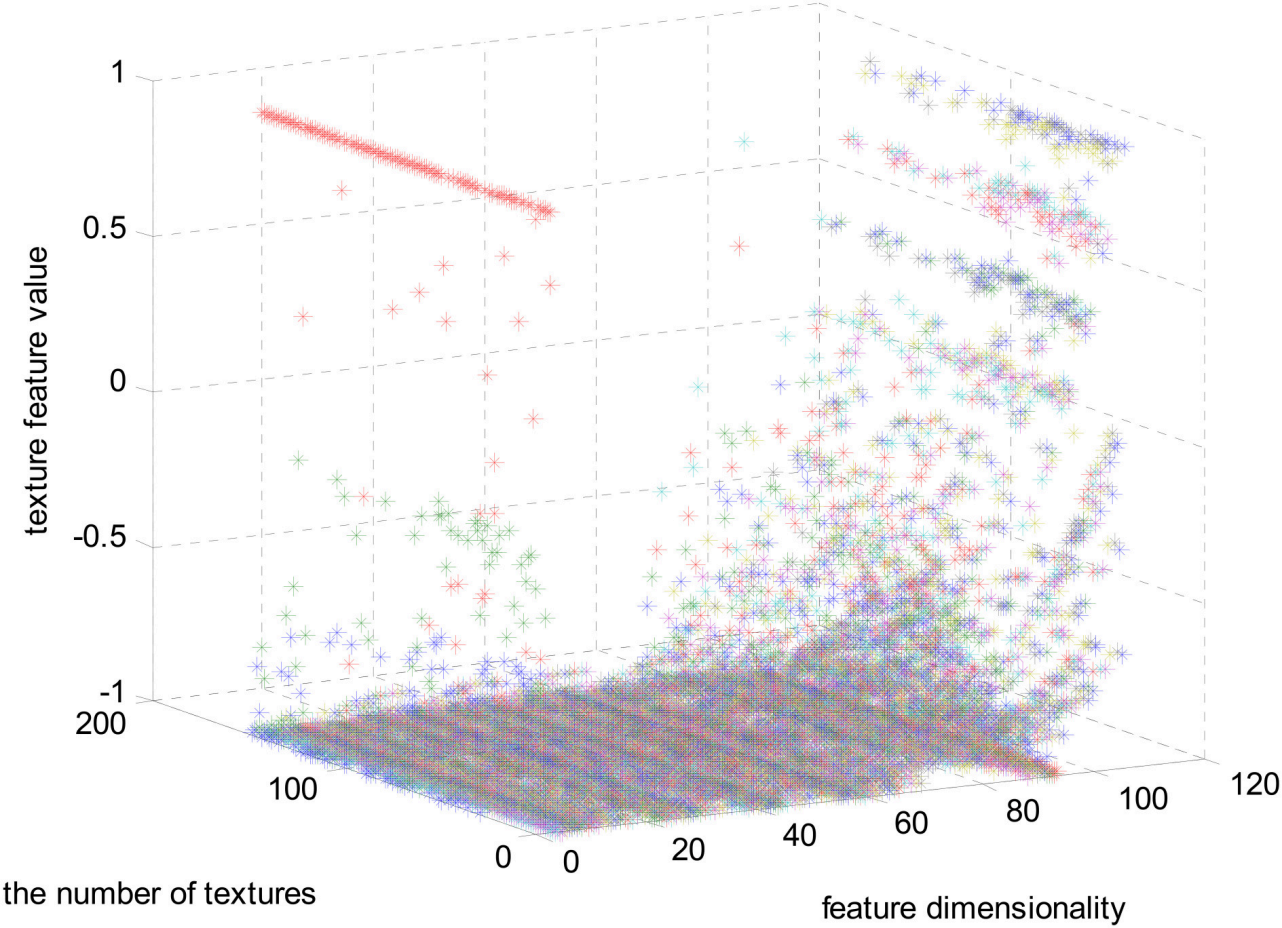
**The full feature matrix comprising 106 features and 151 textures**.

The magnitudes of the features extracted using the algorithms mentioned in Section Materials and Methods are different. In Figure 3, the feature set after normalizing to the interval [−1, 1] using feature scaling method is visualized. In our experiment, 151 selected visual textures are used and 106 features are calculated for each visual texture. So, the size of the feature database is 151 × 106.

**Figure 4:**
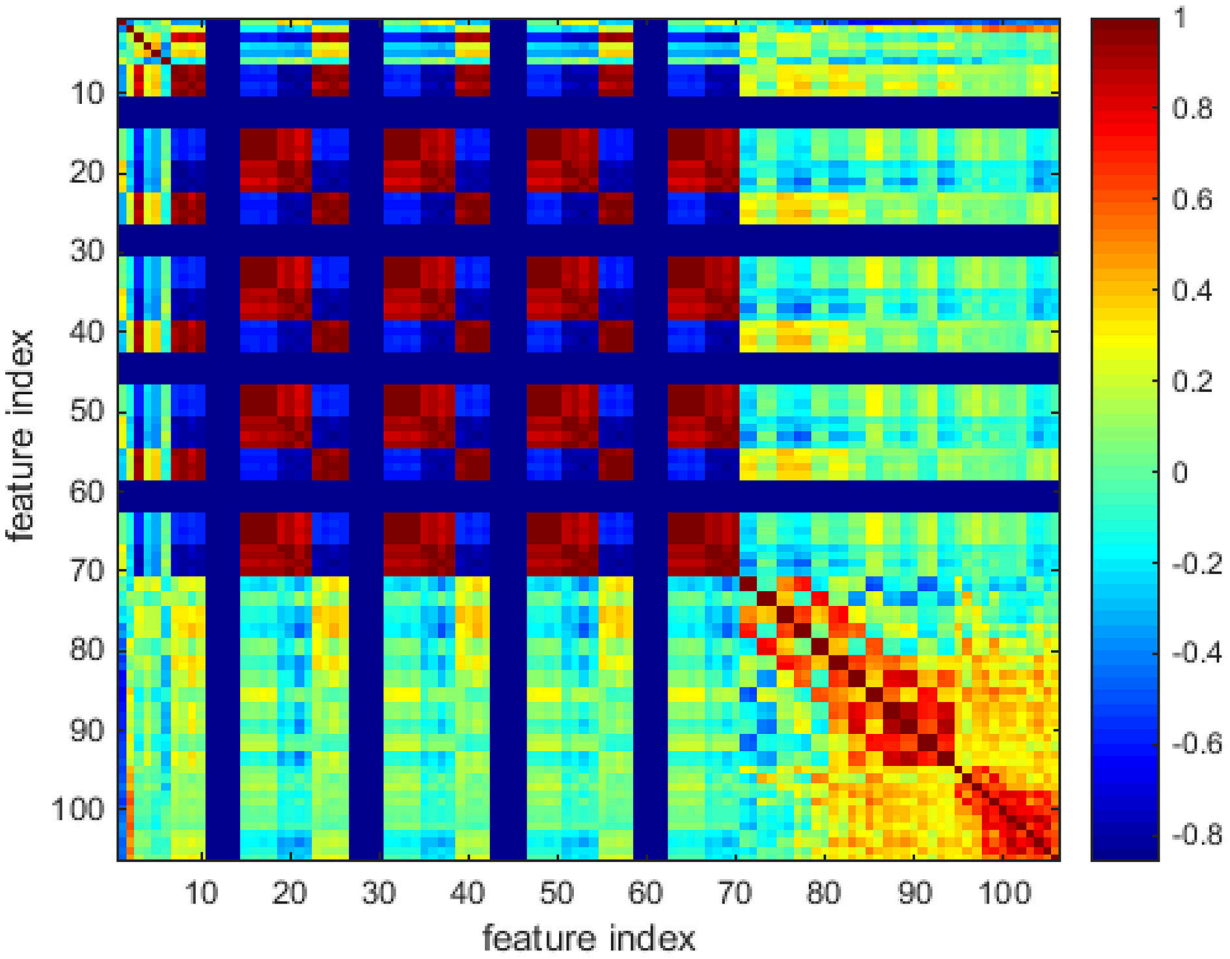
**The colored cross correlation coefficients matrix**.

In Figure 3, one color represents each type of features that locate in each dimensionality. We can find that the majority of the feature values compactly locates at the bottom of the space and only a few sparsely scatter among the concentrated feature stripes. One possible conclusion is that the features extracted using the algorithms mentioned in Section Materials and Methods are highly redundant, correlative and there is a quite low diversity of the features. In order to further examine this issue, the cross correlation coefficients of the 106-D features are calculated and illustrated in Figure 4. There are 1370 correlation coefficients that are larger than 0.75 in their absolute values, which accounts for 12.19% in total.

**Figure 5:**
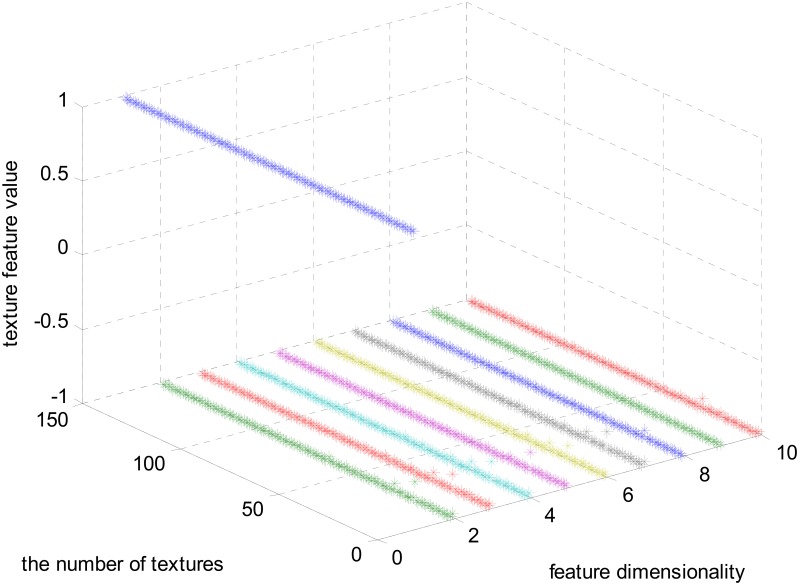
**The first 10 selected features**.

The first 10 features are visualized in Figure 5.

In Figure 5, the normalized 10 features regularly locate in the feature space. The features in the first feature vector, are much larger than the left ones. We also found that the first 10 selected feature vectors can be divided into two clusters, which locate on two poplars of the feature space. The structural risk and the computation complexity of the model will be under constraints through controlling the number of features that are used as inputs. Thus, in the model building process, the features with a Laplacian score lower than 0.85 are not used, which means that we used the first 10 features to build the aesthetic perception model.

### Building a model of aesthetic perception

Below, we discuss model building by means of Eureqa Desktop. Eureqa is a tool that uses a recent breakthrough in machine learning to unpick intrinsic relationships within complex data and explains them as simple mathematical formulas (Schmidt and Lipson, 2009). When the target expressions are defined by Equations (8–10), the basic, trigonometric and exponential functions are selected in the formula building blocks of Eureqa Desktop. In detail, the basic functions include addition, subtraction, multiplication, division and the constant operation. The trigonometric functions include sine, cosine and tangent functions. The exponential functions include exponential, natural logarithmic, factorial, power and square-root functions.

Before model building, the 10 selected features and emotion values were smoothed, outliers removed and normalized with the default algorithms embedded in Eureqa Desktop. The 151 visual textures were divided into two sets. One set is for model building and the other is for model test. The training set included 90% of the total number of textures, and was used for model building. The test set was used to evaluate the performance of the models built on the training set, to measure the expected quality on new textures.

We used a parameter called R^^2^ goodness of fit to evaluate the quantitative goodness of fit between each model and the used data. The model with the greatest R^^2^ is considered to be the best. The models thus selected for the eight pairs of aesthetics properties distributed in the hierarchical feed-forward model are:

(11) $$\begin{array}{l} G(1)=0.03\;\cdot \;f_{1}+598.16\;\cdot \;f_{3}-234.19\;\cdot \;f_{5}-348.67\;\cdot \;f_{7}\cdot \cdot \cdot \\ \;\cdot \cdot \cdot +189.82\;\cdot \;f_{9}-304.29\;\cdot \;f_{10}+22.17 \end{array}$$

(12) $$\begin{array}{r} G\left(2\right)=-1.31\times 10^{-13}\cdot f_{10}-1.73 \end{array}$$

(13) $$\begin{array}{l} G(3)=0.29\;\cdot \;f_{1}+83.14\;\cdot \;f_{7}-53.82\;\cdot \;f_{5}-214.60\;\cdot \;f_{9}\cdot \cdot \cdot \\ \;\cdot \cdot \cdot -0.34\;\cdot \;f_{1}\;\cdot \;f_{7}+216.71\;\cdot \;f_{9}^{\wedge 2}+19.22 \end{array}$$

(14) $$\begin{array}{l} T(1)=0.03\;\cdot \;f_{1}+119.55\;\cdot \;f_{6}-106.46\;\cdot \;f_{7}+18.21\;\cdot \;f_{9}\\ \;-\;51.51\;\cdot \;f_{10}+0.76\;\cdot \;G(3)+2.86 \end{array}$$

(15) $$\begin{array}{l} T(2)=0.09\;\cdot \;f_{1}+691.81\;\cdot \;f_{3}-737.98\;\cdot \;f_{4}-609.32\;\cdot \;f_{6}\\ \;+\;683.64\;\cdot \;f_{7}\cdot \cdot \cdot -\;66.11\;\cdot \;f_{8}+0.33G(3)-9.60 \end{array}$$

(16) $$\begin{array}{l} T(3)=1135.64\;\cdot \;f_{2}-1123.31\;\cdot \;f_{3}-582.81\;\cdot \;f_{4}+571.40\;\cdot \;f_{7}\cdot \cdot \cdot \\ \;\cdot \cdot \cdot -364.15\;\cdot \;f_{8}+376.91\;\cdot \;f_{9}-14.60 \end{array}$$

(17) $$\begin{array}{l} T(4)=185.17\;\cdot \;f_{2}+68.26\;\cdot \;f_{3}-215.02\;\cdot \;f_{4}-99.79\;\cdot \;f_{8}\cdot \cdot \cdot \\ \;\cdot \cdot \cdot +129.36\;\cdot \;f_{9}-0.15\;\cdot \;G(1)+24.37 \end{array}$$

(18) $$\begin{array}{l} Q=123.09\;\cdot \;f_{2}-144.83\;\cdot \;f_{3}-113.49\;\cdot \;f_{8}-148.05\;\cdot \;f_{9}\cdot \cdot \cdot \\ \;\cdot \cdot \cdot +0.64\;\cdot \;T(1)+0.12\;\cdot \;T(3)+1.14 \end{array}$$

where *f*_*i*_,*i* = 1, 2···10 represents the 10 features selected using the Laplacian score algorithm.

In fact, 13 different non-linear terms are chosen for model building in Eureqa, which automatically selects those terms which are most feasible for establishing a high quality model (within a cross-validation procedure). During cross-validation, the training set is split into different folds, and always a separate test fold is used to elicit the error for each training fold combination. According to Hastie et al. (2009), CV is a good method to estimate the expected prediction error on future samples well. Furthermore, in order to overcome over-fitting, we studied how the models listed above performed on a separate test set. We should note the number of variables used to build each model is different. In detail, an input dimensionality of 10 in case of G(1) to G(3), of 13 in case of T(1) to T(4) and of 17 in case of Q.

Surprisingly, we found out that for all models linear terms were sufficient to reach the highest possible quality in terms of R^^2^ goodness of fit for explaining the targets. The exception was for the model for G(3), which uses two quadratic terms. However, these do not boost the quality of this model (cf. Table 3). This is the most noteworthy results of our experiment, as it keeps the model complexity low and thus emphasizes high interpretability capability. Even though the low-level texture features were integrated using non-linear models, the models bridging the gap between computational texture features and aesthetic texture properties turned out to be linear. Additionally, Equations (14–18) indicate that the higher level aesthetic properties in the judgment layer and emotional layers cover—with the exception of the texture features—the aesthetic properties in the lower-level layer. Interestingly, G(3) is an important adjective in the models for T(1) and T(2), whereas T(1) and T(3) have a direct influence on the “like-dislike” feeling.

**Table 3:** **Statistical measures and qualities of models on the training data set (CV-based), the results *after the slashes* correspond to the results reported in (Thumfart et al., 2011) (if available), we offer two additional models for disordered-harmonious (T(4)) and dark-light (G(3))**.

**Aesthetic property**	**R^^2^**	**Basic complexity**	**Enhanced complexity**	**Correlation coefficient**	**RMSE**
G(1)	0.57	6/3	23	0.80	07.5/8.87
G(2)	1.00	1/6	5	1.00	00.00/7.83
G(3)	0.28	6	17	0.44	10.80
T(1)	0.92	6/12	19	0.97	02.42/05.02
T(2)	0.84	7/5	25	0.93	04.31/04.81
T(3)	0.82	6/6	23	0.91	03.39/04.56
T(4)	0.47	6	29	0.93	1.53
Q	0.95	6/6	23	0.98	01.55/03.35

The R^^2^ goodness of fit values of the eight models [shown in Equations (11–18)], are listed in Table 3. Complexity, correlation coefficient and the root mean squared error are also provided to fully evaluate goodness of fit and predictive power. Complexity is important to measure the model's capability in terms of interpretability because of higher complex models are always suffering from interpretability. The root mean squared error shows the expectation deviation between observed and predicted aesthetic property values. Correlation coefficient denotes the correlation between predicted and observed values. Thus, a value close to 1 indicates a nearly perfect prediction; usually, a value of 0.5 and below denotes a useless model. Eureqa's complexity metric (or size) is measured by the number of variables and the relative weights of each of the building blocks used in the solution. This is referred to as “enhanced complexity” in Table 3. Additionally, we report the basic complexity, which is simply the number of input terms in each model. These values are directly comparable with the values in Thumfart et al. (2011) and are directly related to the transparency and understandability of the model (a model with 100 terms can be hard to read and understood, for instance).

In Table 3, the R^^2^ (goodness of fit) for *G*(2), *T*(1), *T*(2), *T*(3), and *Q* are greater than 0.8. In other words, the models for *G*(2), *T*(1), *T*(2), *T*(3), and *Q* are instantiated that provide suitable representations of the aesthetic perceptions. However, it can be seen that the R^^2^ goodness of fit values for *G*(1), *T*(4) and particularly *G*(3) are obviously lower than those of the other models. And, the MSEs of *G*(1) and *G*(3) are significantly greater when compared with the others. The models for T(1) and Q are fully useable and highly precise in case when real G(3) values are available for new textures. Another finding is that our new models based on specifically selected features can significantly outperform the models proposed by Thumfart in terms of prediction error (much lower MSE values).

Note that in model training and evaluation cycles, we always used the original data gotten from the semantic differential. In particular, for establishing a model for T(1) and Q, which both use G(3) as input, the original G(3) values from the interview data were used and not the predictions of the G(3) model (which were particularly poor as can be seen in Table 3). Model building and the final models for T(1) and Q were therefore not affected.

**Figure 6:**
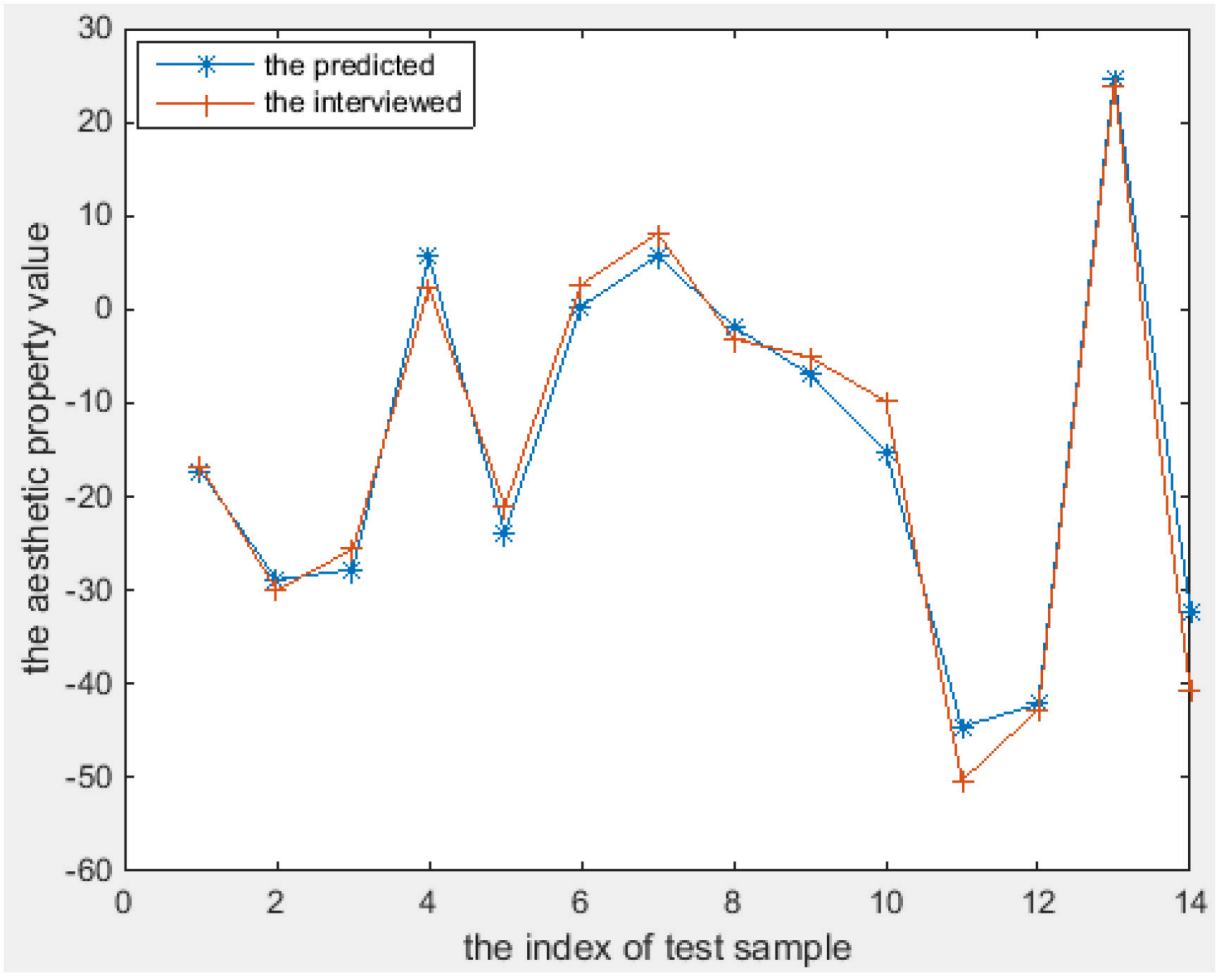
**The predicted and the interviewed test sample values for T(3)**.

**Figure 7:**
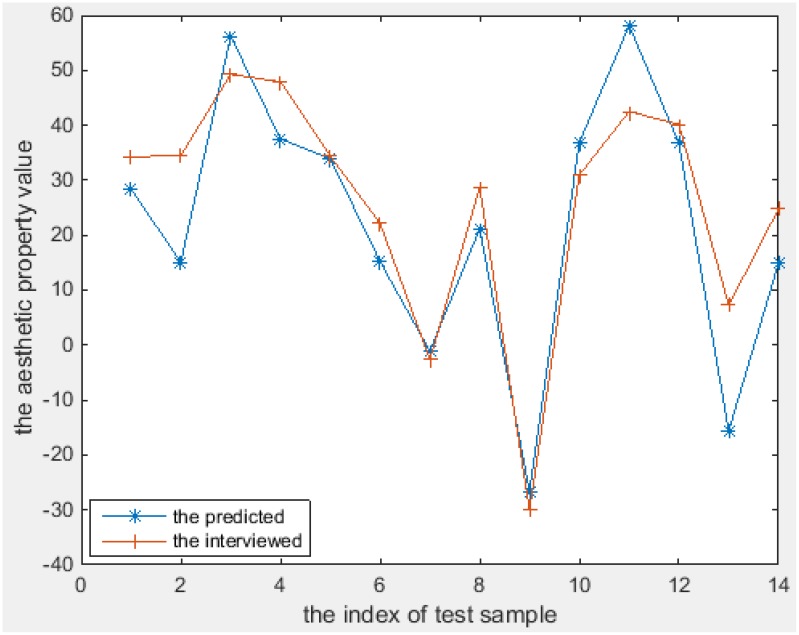
**The predicted and the interviewed test sample values for T(4)**.

**Figure 8:**
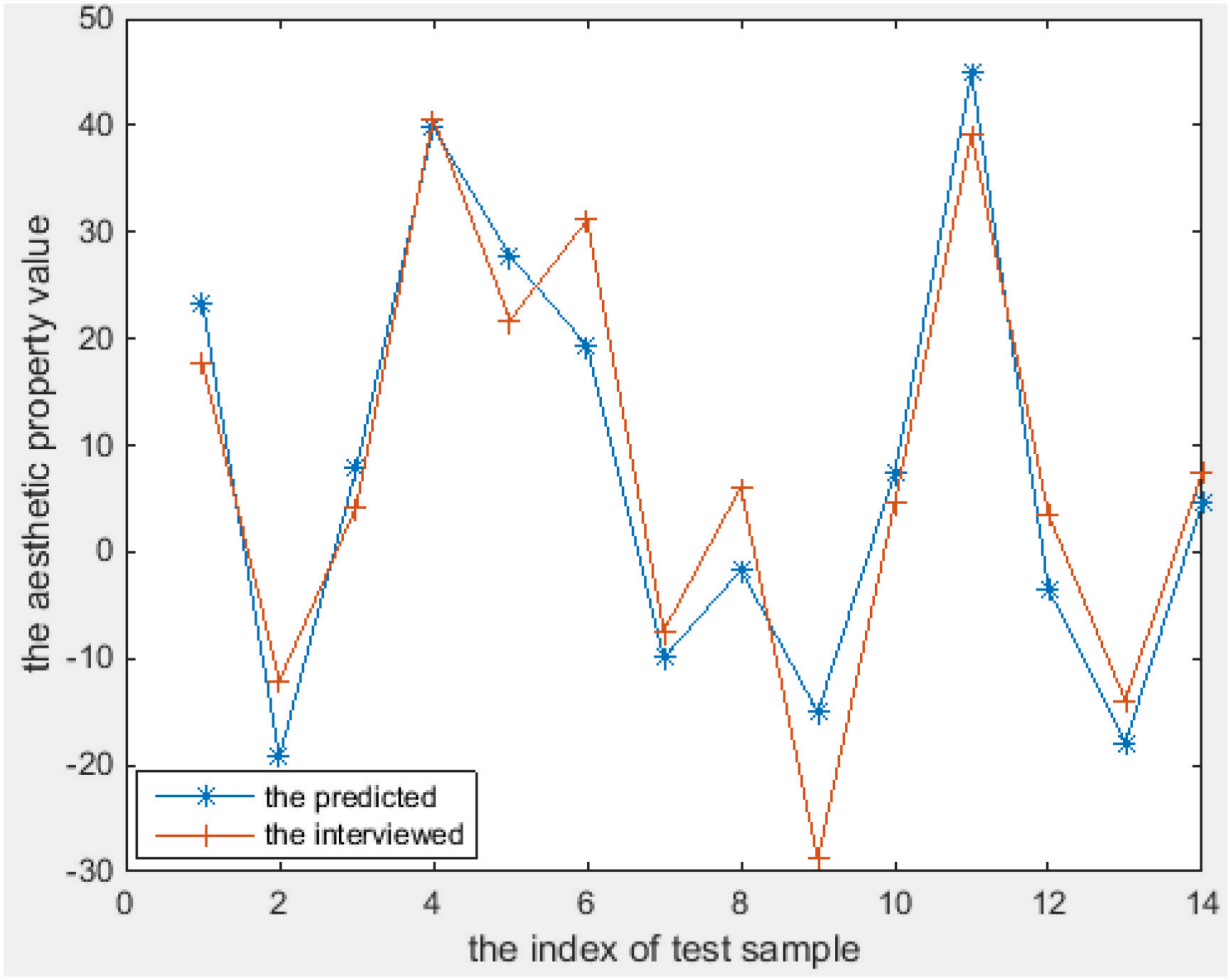
**The predicted and the interviewed test sample values for Q**.

The aesthetics properties predicted using these models [according to Equations (11–18)] and the values from the interview-based test set that comprises 14 textures are plotted in Figures 6–8 for the three most interesting and challenging properties “artificial-natural,” “disordered-harmonious,” and “like-dislike.” The statistical measures of the predicted and real aesthetic property values from interviews for the test set are given in Table 4.

**Table 4:** **Statistical measures for the test set**.

**Aesthetic property**	**Correlation coefficient**	**RMSE**
G(1)	0.99	1.74
G(2)	0.99	1.13
G(3)	0.76	9.28
T(1)	0.99	1.79
T(2)	0.99	2.51
T(3)	0.98	3.77
T(4)	0.94	5.68
Q	0.97	4.73

As shown in Figures 6–8, the prediction power of G(1), G(2), T(1), T(2), and T(3) is better than that of G(3), T(4), and Q. However, the predictive power of G(3) and T(4) is much better on the test set than on the training set, at least for T(4). We therefore conclude that the models can be used to calculate the properties of textures. In fact, the maximum correlation coefficient of the training set is greater than that of the test set in all cases, as can be seen in Table 3, 4. In other words, the multiple linear regression models can predict the aesthetic property well for some new visual textures that were not used in the training stage, even though the correlation coefficient on the overall training set is unsatisfactory. Another indication is that the bias is higher than the variance error, and thus over-fitting does not take place. On the other hand, we could find out that also the models for G(3) and T(4) can perform well on a subset of the whole texture set, which makes them promising for other textures collected in the future.

## Conclusions

In this paper, we have proposed a hierarchical feed-forward layer structure built by multiple linear regression to investigate the relationship between human aesthetic texture perception and computational low-level texture features. Rather than black-box models, we sought to build nearly white-box models that can be interpreted both in terms of structure and interrelations between aesthetic properties and texture features according to feature weights.

First, we carried out a texture analysis and calculated 106 color and texture features for each visual texture. To achieve the best possible prediction rate and reduce the complexity of model building, feature selection using the Laplacian Score was employed to choose the best feature subset (finally comprising 10 features). Then, the aesthetic properties of a set of 151 visual textures were collected in a semantic differential experiment with 20 subjects. Eight pairs of antonyms were selected to describe aesthetic properties for emotion perception in different affective layers. Finally, we utilized multiple regression techniques employing a variety of functional terms to bridge the gap between computational texture features and aesthetic emotions in form of mappings within a hierarchical layered structure model.

The best model for each of the 8 aesthetic properties (except for the “dark-light” pair) is a linear function, even though non-linear terms were selected in Eureqa Desktop when models are initialized. Furthermore, these built models are in low dimensionality. In other words, the models only use a quite low number of terms, namely 7 maximal, and in most cases 6. This is helpful to the readability, interpretability and understandability for psychologists. The 8 models have lower errors than the models designed in Thumfart et al. (2011) for all aesthetic properties, which confirms the feasibility and applicability of our models in future works. Additionally, the experiment indicates that—with the exception of texture features—the higher level aesthetic properties in the judgment and emotional layers cover the aesthetic properties in the lower-level layer.

As part of future work, we will select more visual texture samples and include more subjects in the semantic differential experiment, especially to investigate the influences of the types of features and functions selected for model building. This should help to improve the lower quality models, especially that built for G(3). Additional future work will include:

1. Considering more complex non-linear regression modeling architectures (rather than plain transformations), especially regression trees and/or fuzzy systems, which both offer interpretability from another viewpoint. Their structures are readable as IF-THEN rules and provide better insights into the relations between input features and targets.
2. Perception modeling that considers different groups of people, e.g., a gender study or a study with respect to age, education etc.: the interview data is split into different groups and a model is created for each group. This could provide interesting answers to questions such as “Do women or men rate textures more consistently?” or “Do women or men trigger creation of different models?”

### Conflict of interest statement

The authors declare that the research was conducted in the absence of any commercial or financial relationships that could be construed as a potential conflict of interest.

## References

[B1] Augello A. Infantino I. Pilato G. Rizzo R. Vella F. (2013). Binding representational spaces of colors and emotions for creativity. Biol. Inspired Cogn. Archit. 5, 64–71. 10.1016/j.bica.2013.05.005

[B2] Axelsson Ö. (2007). Individual differences in preferences to photographs.pdf. Psychol. Aesthetics Creat. Arts 1, 61–72. 10.1037/1931-3896.1.2.61

[B3] Baraldi A. Parmiggiani F. (1995). An investigation of the textural characteristics associated with gray level cooccurrence matrix statistical parameters. IEEE Trans. Geosci. Remote Sens. 33, 293–304. 10.1109/36.377929

[B4] Bharati M. H. Liu J. J. MacGregor J. F. (2004). Image texture analysis: methods and comparisons. Chemom. Intell. Lab. Syst. 72, 57–71. 10.1016/j.chemolab.2004.02.005

[B5] Breiman L. Friedman J. Stone C. J. Olshen R. A. (1993). Classification and Regression Trees. Boca Raton, FL: Chapman and Hall.

[B6] Brooks R. R. Grewe L. Iyengar S. S. (2001). Recognition in the wavelet domain: a survey. J. Electron. Imaging 10, 757–784. 10.1117/1.1381560

[B7] Bundgaard P. F. (2014). Feeling, meaning, and intentionality—a critique of the neuroaesthetics of beauty. Phenomenol. Cogn. Sci. 10.1007/s11097-014-9351-5 Available online at: http://link.springer.com/article/10.1007/s11097-014-9351-5

[B8] Castelli V. Bergman L. D. (2002). Image Databases: Search and Retrieval of Digital Imagery. The Second. New York, NY: JOHN Wiley & SONS, INC.

[B9] Cela-Conde C. J. García-Prieto J. Ramasco J. J. Mirasso C. R. Bajo R. Munar E.. (2013). Dynamics of brain networks in the aesthetic appreciation. Proc. Natl. Acad. Sci. U.S.A. 110, 10454–10461. 10.1073/pnas.1302855110 23754437 PMC3690613

[B10] Cela-Conde C. J. Agnati L. Huston J. P. Mora F. Nadal M. (2011). The neural foundations of aesthetic appreciation. Prog. Neurobiol. 94, 39–48. 10.1016/j.pneurobio.2011.03.003 21421021

[B11] Chandrashekar G. Sahin F. (2014). A survey on feature selection methods. Comput. Electr. Eng. 40, 16–28. 10.1016/j.compeleceng.2013.11.024

[B12] Chatterjee A. Vartanian O. (2014). Neuroaesthetics. Trends Cogn. Sci. 18, 370–375. 10.1016/j.tics.2014.03.003 24768244

[B13] Chuang Y. Chen L. (2008). How to rate 100 visual stimuli efficiently. Int. J. Des. 2, 31–43.

[B14] Datta R. Joshi D. Li J. Wang J. (2006). Studying aesthetics in photographic images using a computational approach. Lect. Notes Comput. Sci. 3953, 288–301. 10.1007/11744078_23

[B15] Davis L. S. Johns S. A. Aggarwal J. K. (1979). Texture analysis using generalized co-occurrence matrices. IEEE Trans. Pattern Anal. Mach. Intell. 1, 251–259. 10.1109/TPAMI.1979.4766921 21868856

[B16] Do M. N. Vetterli M. (2002). Wavelet-based texture retrieval using generalized Gaussian density and Kullback-Leibler distance. IEEE Trans. Image Process. 11, 146–158. 10.1109/83.982822 18244620

[B17] Dong Y. Ma J. (2011). Wavelet-based image texture classification using local energy histograms. Signal Process. Lett. IEEE 18, 247–250. 10.1109/LSP.2011.2111369

[B18] Elkharraz G. Thumfart S. Akay D. Eitzinger C. Henson B. (2014). Making tactile textures with predefined affective properties. IEEE Trans. Affect. Comput. 5, 57–70. 10.1109/T-AFFC.2013.21

[B19] Graham D. J. Meng M. (2011). Artistic representations: clues to efficient coding in human vision. Vis. Neurosci. 28, 371–379. 10.1017/S0952523811000162 21838937

[B20] Groissboeck W. Lughofer E. Thumfart S. (2010). Associating visual textures with human perceptions using genetic algorithms. Inf. Sci. 180, 2065–2084. 10.1016/j.ins.2010.01.035

[B21] Guo X. Asano C. Asano A. Kurita T. Li L. (2012). Analysis of texture characteristics associated with visual complexity perception. Opt. Rev. 19, 306–314. 10.1007/s10043-012-0047-1

[B22] Guyon I. Elisseeff A. (2003). An introduction to variable and feature selection. J. Mach. Learn. Res. 3, 1157–1182.

[B23] Hanada M. (2013). Analyses of color emotion for color pairs with independent component analysis and factor analysis. Color Res. Appl. 38, 297–308. 10.1002/col.20750

[B24] Haralick R. Shanmugam K. Dinstein I. (1973). Textural features for image classification. IEEE Trans. Syst. Man Cybern. 3, 610–621. 10.1109/TSMC.1973.4309314

[B25] Hastie T. Tibshirani R. Friedman J. (2009). The Elements of Statistical Learning: Data Mining, Inference and Prediction, 2nd Edn. New York, NY; Berlin; Heidelberg: Springer.

[B26] He X. Cai D. Niyogi P. (2005). Laplacian score for feature selection, in Advances in Neural Information Processing Systems, Vol. 18 (NIPS'05) (Cambridge, MA; London: MIT Press), 1–8.

[B27] Ishizu T. Zeki S. (2013). The brain's specialized systems for aesthetic and perceptual judgment. Eur. J. Neurosci. 37, 1413–1420. 10.1111/ejn.12135 23373763 PMC3792471

[B28] Jiang W. Loui A. C. Cerosaletti C. D. (2010). Automatic aesthetic value assessment in photographic images, in 2010 IEEE International Conference on Multimedia and Expo (Suntec City: IEEE), 920–925.

[B29] Karu K. Jain A. Bolle R. (1996). Is there any texture in the image? Pattern Recognit. 29, 1437–1446. 10.1016/0031-3203(96)00004-024841370

[B30] Leder H. Belke B. Oeberst A. Augustin D. (2004). A model of aesthetic appreciation and aesthetic judgments. Br. J. Psychol. 95, 489–508. 10.1348/0007126042369811 15527534

[B31] Leder H. Gerger G. Dressler S. G. Schabmann A. (2012). How art is appreciated. Psychol. Aesthetics Creat. Arts 6, 2–10. 10.1037/a0026396

[B32] Levinson J. (2006). Contemplating Art. Oxford: Oxford Scholarship Online Monographs.

[B33] Liu J. Zuo B. Zeng X. Vroman P. Rabenasolo B. (2011). Expert Systems with Applications Wavelet energy signatures and robust Bayesian neural network for visual quality recognition of nonwovens. Expert Syst. Appl. 38, 8497–8508. 10.1016/j.eswa.2011.01.049

[B34] Ljung L. (1999). System Identification: Theory for the User. New Jersey, NJ: Prentice Hall PTR; Prentic Hall Inc.; Upper Saddle River.

[B35] Lucassen M. P. Gevers T. Gijsenij A. (2011). Texture affects color emotion. Color Res. Appl. 36, 426–436. 10.1002/col.20647

[B36] Lughofer E. (2011). Evolving Fuzzy Systems—Methodologies, Advanced Concepts and Applications. Berlin; Heidelberg: Springer.

[B37] Man D. Wei D. Chih-Chieh Y. (2013). Product color design based on multi-emotion. J. Mech. Sci. Technol. 27, 2079–2084. 10.1007/s12206-013-0518-8

[B38] Manfredi L. R. Saal H. P. Brown K. J. Zielinski M. C. Dammann J. F. III. Polashock V. S.. (2014). Natural scenes in tactile texture. J. Neurophysiol. 111, 1792–1802. 10.1152/jn.00680.2013 24523522

[B39] Ou L.-C. Luo M. R. Woodcock A. Wright A. (2004a). A study of colour emotion and colour preference. Part I: colour emotions for single colours. Color Res. Appl. 29, 232–240. 10.1002/col.20010

[B40] Ou L.-C. Luo M. R. Woodcock A. Wright A. (2004b). A study of colour emotion and colour preference. Part II: colour emotions for two-colour combinations. Color Res. Appl. 29, 292–298. 10.1002/col.20024

[B41] Ou L.-C. Ronnier Luo M. Sun P.-L. Hu N.-C. Chen H.-S. Guan S.-S. (2012). A cross-cultural comparison of colour emotion for two-colour combinations. Color Res. Appl. 37, 23–43. 10.1002/col.20648

[B42] Palmer S. E. Schloss K. B. Sammartino J. (2013). Visual aesthetics and human preference. Annu. Rev. Psychol. 64, 77–107. 10.1146/annurev-psych-120710-100504 23020642

[B43] Roberti F. Siqueira D. Robson W. Pedrini H. (2013). Multi-scale gray level co-occurrence matrices for texture description. Neurocomputing 120, 336–345. 10.1016/j.neucom.2012.09.042

[B44] Romani S. Sobrevilla P. Montseny E. (2012). Variability estimation of hue and saturation components in the HSV space. Color Res. Appl. 37, 261–271. 10.1002/col.20699

[B45] Schmidt M. Lipson H. (2009). Distilling free-form natural laws from experimental data. Science 324, 81–85. 10.1126/science.1165893 19342586

[B46] Simmons D. R. (2012). Colour and emotion, in New Directions in Colour Studies, eds Biggam C. P. Hough C. A. Kay C. J. Simmons D. R. (Amsterdam: John Benjamins), 395–414.

[B47] Skedung L. Arvidsson M. Chung J. Y. Stafford C. M. Berglund B. Rutland M. W. (2013). Feeling small: exploring the tactile perception limits. Sci. Rep. 3, 1–6. 10.1038/srep02617 24030568 PMC3771396

[B48] Tabakhi S. Moradi P. Akhlaghian F. (2014). An unsupervised feature selection algorithm based on ant colony optimization. Eng. Appl. Artif. Intell. 32, 112–123. 10.1016/j.engappai.2014.03.007

[B49] Tamura H. Mori S. Yamawaki T. (1978). Textural features corresponding to visual perception. Syst. Man Cybern. IEEE Trans. 75, 460–473. 10.1109/TSMC.1978.4309999

[B50] Thumfart S. Heidl W. Scharinger J. Eitzinger C. (2009). A quantitative evaluation of texture feature robustness and interpolation behaviour, in Proceedings of The 13th International Conference on Computer Analysis of Images and Patterns (Münster), 1154–1161.

[B51] Thumfart S. Jacobs R. Lughofer E. Eitzinger C. Cornelissen F. W. Groissboeck W. (2011). Modeling human aesthetic perception of visual textures. ACM Trans. Appl. Percept. 8, 1–29. 10.1145/2043603.2043609

[B52] Toet A. Henselmans M. Lucassen M. P. Gevers T. (2011). Emotional effects of dynamic textures. Iperception 2, 969–991. 10.1068/i0477 23145257 PMC3485791

[B53] Vapnik V. (1998). Statistical Learning Theory. New York, NY: John Wiley & Sons.

[B54] Zeki S. (2002). Trying to make sense of art. Nature 418, 918–919. 10.1038/418918a

